# NREM sleep as a novel protective cognitive reserve factor in the face of Alzheimer's disease pathology

**DOI:** 10.1186/s12916-023-02811-z

**Published:** 2023-05-03

**Authors:** Zsófia Zavecz, Vyoma D. Shah, Olivia G. Murillo, Raphael Vallat, Bryce A. Mander, Joseph R. Winer, William J. Jagust, Matthew P. Walker

**Affiliations:** 1 Department of Psychology, Center for Human Sleep Science, University of California Berkeley, Berkeley, CA 94720 USA; 2 Department of Psychiatry and Human Behavior, University of California, Irvine, CA 92617 USA; 3 Department of Neurology and Neurological Sciences, Stanford University School of Medicine, Stanford, CA 94304 USA; 4 Helen Wills Neuroscience Institute, University of California Berkeley, Berkeley, CA 94720 USA; 5 Molecular Biophysics and Integrated Bioimaging, Lawrence Berkeley National Laboratory, Berkeley, CA 94720 USA

**Keywords:** Alzheimer’s disease, β-amyloid pathology, Memory, Sleep, Slow wave activity, Cognitive reserve, Resilience

## Abstract

**Background:**

Alzheimer’s disease (AD) pathology impairs cognitive function. Yet some individuals with high amounts of AD pathology suffer marked memory impairment, while others with the *same* degree of pathology burden show little impairment. Why is this? One proposed explanation is cognitive reserve i.e., factors that confer resilience against, or compensation for the effects of AD pathology. Deep NREM slow wave sleep (SWS) is recognized to enhance functions of learning and memory in healthy older adults. However, that the quality of NREM SWS (NREM slow wave activity, SWA) represents a novel cognitive reserve factor in older adults with AD pathology, thereby providing compensation against memory dysfunction otherwise caused by high AD pathology burden, remains unknown.

**Methods:**

Here, we tested this hypothesis in cognitively normal older adults (*N* = 62) by combining ^11^C-PiB (Pittsburgh compound B) positron emission tomography (PET) scanning for the quantification of β-amyloid (Aβ) with sleep electroencephalography (EEG) recordings to quantify NREM SWA and a hippocampal-dependent face-name learning task.

**Results:**

We demonstrated that NREM SWA significantly moderates the effect of Aβ status on memory function. Specifically, NREM SWA selectively supported superior memory function in individuals suffering high Aβ burden, i.e., those most in need of cognitive reserve (*B* = 2.694, *p* = 0.019). In contrast, those without significant Aβ pathological burden, and thus without the same  need for cognitive reserve, did not similarly benefit from the presence of NREM SWA (*B* = -0.115, *p* = 0.876). This interaction between NREM SWA and Aβ status predicting memory function was significant after correcting for age, sex, Body Mass Index, gray matter atrophy, and previously identified cognitive reserve factors, such as education and physical activity (*p* = 0.042).

**Conclusions:**

These findings indicate that NREM SWA is a novel cognitive reserve factor providing resilience against the memory impairment otherwise caused by high AD pathology burden. Furthermore, this cognitive reserve function of NREM SWA remained significant when accounting both for covariates, and factors previously linked to resilience, suggesting that sleep might be an independent cognitive reserve resource. Beyond such mechanistic insights are potential therapeutic implications. Unlike many other cognitive reserve factors (e.g., years of education, prior job complexity), sleep is a modifiable factor. As such, it represents an intervention possibility that may aid the preservation of cognitive function in the face of AD pathology, both present moment and longitudinally.

**Supplementary Information:**

The online version contains supplementary material available at 10.1186/s12916-023-02811-z.

## Background

The prevalence of Alzheimer’s disease (AD) shows an almost exponential increase with age (currently averaging around 10% in individuals above the age of 65), causing the number of individuals affected by it to escalate as life expectancy increases [1, 2]. Further, Alzheimer’s disease and its associated pathologies of β-amyloid (Aβ) and tau are typified by cognitive impairment [3–10]. Consistent with this, individuals with high Aβ burden (Aβ positive, Aβ+) have a worse cognitive function and undergo faster cognitive decline than those with low Aβ burden (Aβ negative, Aβ- [3–6]).

However, a paradox has become increasingly clear: some individuals with high amounts of Aβ pathology suffer marked memory impairment, while others with the *same* degree of pathology burden show little impairment [11]. Why is this? One proposed explanation is cognitive reserve [12, 13], i.e., factors that offer compensation against the effects of substantive AD pathology.

Cognitive reserve factors identified to date include a greater number of years of education [14–16], higher occupational complexity [17, 18], and higher levels of physical activity [19, 20]. Nevertheless, even when combining all reserve factors identified to date, they explain only a modest degree of the full magnitude of cognitive reserve expressed across individuals [21]. This indicates that other such factors must exist that have yet to be identified.

Here, we propose that one novel and currently unexplored factor supporting cognitive reserve in the face of Aβ pathology burden is sleep, and specifically the quality of non-rapid eye movement slow wave sleep (NREM SWS). Five lines of evidence support this hypothesis.

First, a robust literature has demonstrated the beneficial effect of sleep on cognitive performance, particularly for hippocampal-dependent learning and memory [22, 23]. Second, manipulations of NREM SWS and the electroencephalographic (EEG) quality of NREM SWS (indexed in slow wave activity, SWA) causally enhance cognitive function in older adults, and in those with mild cognitive impairment [24, 25]. Third, selective deprivation of NREM SWA in older adults causally impairs hippocampal activity and associated learning, especially for item-based memory [26]. Fourth, Aβ burden is associated with impairments in NREM SWA, which in turn, predicts worse memory performance [27]. Fifth, degraded memory performance is associated with worse sleep efficiency, most prominently in individuals with high Aβ burden [28].

Despite such converging evidence, the possibility that NREM SWA represents a neurophysiological cognitive reserve factor that supports superior memory function under conditions of high Aβ burden remains untested. Of note, the proposal of NREM SWA as a cognitive reserve factor is dissociable from existing findings that show that impaired sleep is associated with worse and declining memory in older adults, and in those with AD pathology. Instead, the current hypothesis describes the opposite scenario: when facing severe AD pathology burden, NREM SWA beneficially mitigates against that high AD pathological state and supports superior cognitive function as a result. That is, we propose a new pathway through which sleep and cognitive function are connected in AD, namely a cognitive reserve pathway wherein NREM SWA confers protective compensation against existing AD pathology burden.

We tested this overarching hypothesis by combining ^11^C-PiB (Pittsburgh compound B) positron emission tomography (PET) scanning, which offers *in vivo* estimates of Aβ burden, with sleep EEG recordings quantifying NREM SWA, and a behavioral test of sleep-dependent hippocampal-related learning, focusing on item-based memory [26]. This design offered a test of the prediction that NREM SWA moderates the effect of Aβ pathology burden on memory function, such that NREM SWA supports superior cognitive performance under circumstances of high need for cognitive reserve, i.e., high Aβ burden. Thus, the study addressed the three formal components that have been described in the characterization of cognitive reserve [12, 13]: 1) a feature or disease measurement known to impact cognition (here, Aβ), 2) a measure of cognition (here, memory function), and 3) a variable that influences the relationship between Aβ and memory (here, NREM SWA).

## Methods

### Participants

**Fig. 1:**
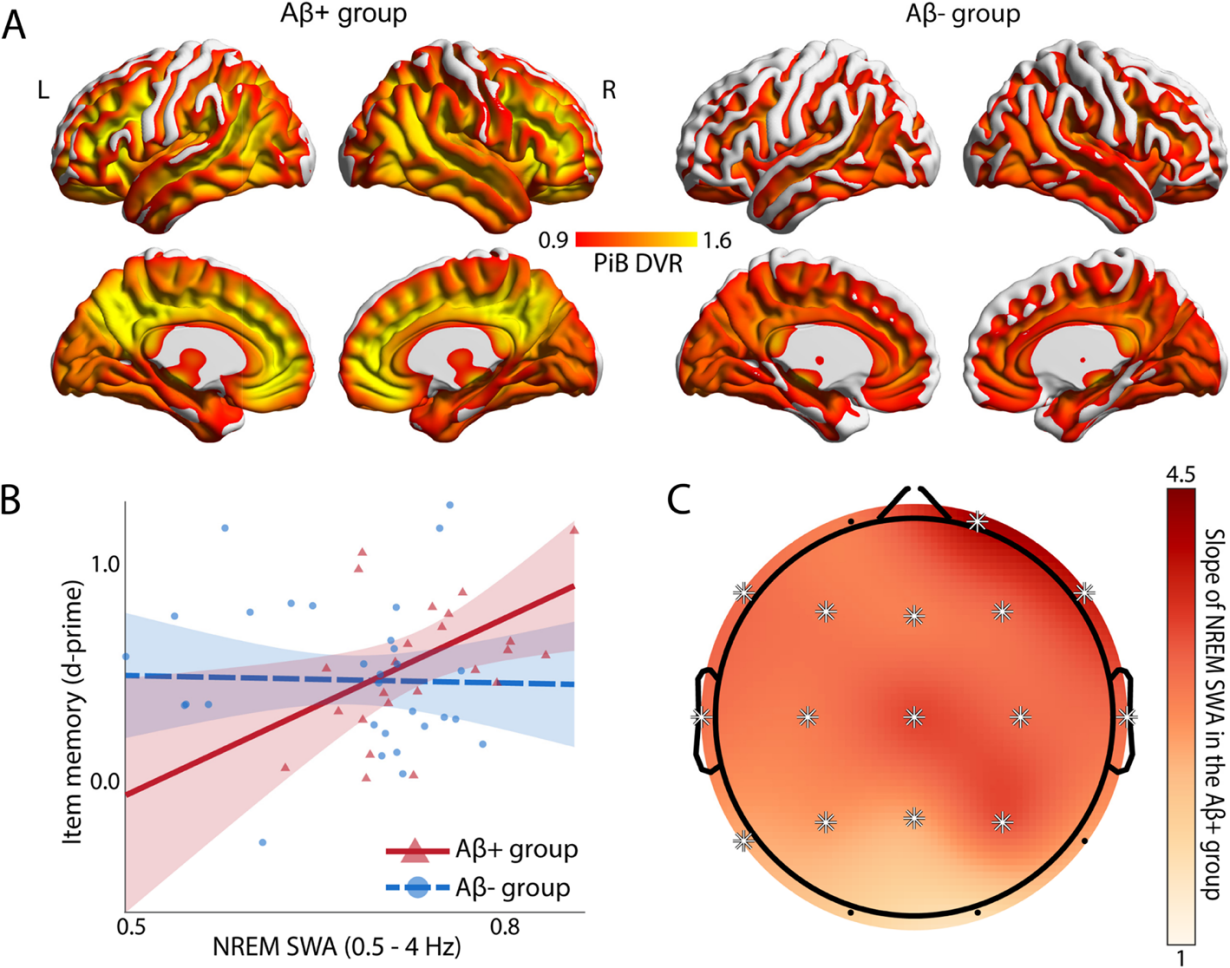
β-amyloid (Aβ) burden and sleep-related cognitive reserve in the Aβ+ and Aβ- groups. **A** Mean voxelwise ^11^C-PiB DVR PET maps in the Aβ+ (left) and Aβ- (right) groups demonstrating Aβ distribution. **B** Association between item memory and NREM SWA averaged across the scalp (indexed by relative delta bandpower) in the Aβ+ (red) and Aβ- (blue) groups after adjusting for age, sex, BMI, gray matter atrophy, education, physical activity, and the time difference between the PET and sleep sessions. NREM SWA supported superior memory function in individuals suffering high Aβ burden, i.e., those most in need of cognitive reserve, and not in those without such pathological abutment needs, i.e., those with low Aβ burden (regression line, 95% confidence interval, and individual subject data points illustrated in red and blue for each respective group). **C** EEG topographic plot of NREM SWA predicting memory function in the Aβ+ group (slopes adjusted for age, sex, BMI, gray matter atrophy, education, physical activity, and the time difference between the PET and sleep sessions). Asterisks indicate FDR corrected *p*s < 0.05. The strength of the associations was comparable over the scalp with the strongest associations observed over right frontal regions

Sixty-two cognitively normal older adults participated in the study. Thirty-one individuals were β-amyloid positive (Aβ+) and thirty-one were Aβ-, based on an established cutoff (1.065 global PiB distribution volume ratio (DVR) [29, 30], and see Table 1 and Fig. 1A). Data from twenty-six of these participants were included in a previous publication [27]. Participants were recruited from the Berkeley Aging Cohort Study (BACS) which allowed for the selection and enrollment of participants with a full range of PiB DVR values. Exclusion criteria were history of neurologic, psychiatric, or sleep disorders, current use of antidepressant or hypnotic medications, and presence of contraindications for exposure to the high magnetic field of MRI imaging. All participants scored ≥ 25 on the Mini-Mental State Examination (MMSE). Several factors motivated the inclusion of cognitively normal elderly individuals in the current study. First, in the early stages of Alzheimer’s disease (AD) pathology, the diversity and severity of different pathologies in the brain are less extensive and therefore more optimal when studying specific pathologies (i.e., Aβ) in isolation, as motivated by the current hypothesis. Second, the early stages of the AD pathological cascade, such as Aβ pathology [10], allow for cognitive reserve to the greatest extent, indicated by a higher proportion of cognitively normal elderly adults with only Aβ pathology than with Aβ pathology and neurodegeneration [34, 35]. Finally, from a treatment perspective, results in such a population allow insights for early therapeutic intervention.

**Table 1:** Demographics, PET, cognitive and sleep summary information (mean ± SD)

Variable	Aβ+ group (*N* = 31)	Aβ- group (*N* = 31)
Age (years)	75.97 ± 3.81	75.26 ± 6.08
Sex (% Female)	68	55
BMI	25.65 ± 3.91^a^	24.74 ± 4.16
Education (years)	16.65 ± 2.06	17.19 ± 1.49
Global PiB DVR	1.34 ± 0.26	1.02 ± 0.03***
Time interval between PET and PSG sessions (years)	0.63 ± 0.06	0.62 ± 0.08
Total sleep time (min)	325 ± 72	330 ± 63
NREM2 time (min)	178 ± 57	192 ± 60
NREM3 time (min)	64 ± 53	52 ± 36
REM time (min)	48 ± 19	48 ± 21
WASO (min)	121 ± 64	121 ± 53
NREM SWA (relative delta power)	0.73 ± 0.07	0.68 ± 0.08*
MMSE	29.13 ± 1.06	28.90 ± 1.19
Item memory (d-prime)	0.56 ± 0.34^b^	0.54 ± 0.41
Associative memory (d-prime)	0.51 ± 0.37^b^	0.46 ± 0.38
Memory composite score (z-score)	-0.11 ± 0.78	-0.13 ± 0.93

In line with previous findings [27], NREM SWA, defined as NREM relative delta power in the 0.5 to 4 Hz frequency range, was significantly higher in the Aβ+ group compared to the Aβ- group (p = 0.01, see section ‘*Association between Aβ burden and spectral power in the delta frequency range’* and Figure S1 in Additional file 1 [27, 31–33])

*BMI* Body Mass Index, *PiB DVR* Pittsburgh compound B Distribution Volume Ratio, *PET* Positron Emission Tomography, *PSG* Polysomnography, *NREM* Non-rapid eye movement, *WASO* Wake after sleep onset, *SWA* Slow wave activity, *MMSE* Mini-Mental State Examination

*p < 0.05

***p < 0.001

^a^*N* = 30

^b^*N* = 28

### General experimental design

All participants completed three sessions. In one session, participants underwent PET scanning following ^11^C-PiB injection to measure Aβ burden. Within 2 years of ^11^C-PiB PET scanning (median: 0.52 years, and see Table 1), participants completed the sleep study session. Participants spent two nights in the laboratory, where they were given 8-hour sleep opportunities monitored with polysomnography (PSG). This 8-hour interval was in accordance with age-appropriate averages [36], and the total sleep time of the participants was in the normative range [37] (Table 1). The first night served as an adaptation night to prevent first-night effects [38, 39], whereas the second served as the experimental night. Following the experimental night, structural MRI scans were obtained from all participants in the morning to measure gray matter atrophy. Subsequently, participants completed a hippocampal-dependent face-name memory task [40]. Four participants had missing data in the memory task due to not completing the task (*N* = 2) and insufficient number of certain response types (*N* = 2) resulting in an inability to compute memory measures (*N* = 1 missing measure for item and *N* = 1 missing measure for associative memory). Furthermore, BMI was not assessed for one participant and structural MRI was missing for one participant. All participants abstained from caffeine, alcohol, and daytime naps for 48 hours before and during the sleep study session. Participants kept habitual sleep-wake rhythms for at least 1 week preceding the in-laboratory sleep session and completed the in-laboratory sleep study in accordance with their individual rhythms. In a separate session (on average within a half year from the experimental sleep session, median: 0.44 years), participants also completed multiple questionnaires and cognitive tasks, including the assessment of physical activity in the last year [41] and a composite memory measure [42].

### PET scanning

^11^C-PiB PET imaging, quantifying Aβ burden, was conducted in 3D acquisition mode using an ECAT EXACT HR (*N* = 20) or BIOGRAPH Truepoint 6 scanner (Siemens Medical Systems, Erlangen, Germany). ^11^C-PiB was synthesized at the Lawrence Berkeley National Laboratory Biomedical Isotope Facility using a published protocol and described in detail previously [43]. After intravenous injection of ∼15 mCi of ^11^C-PiB into the antecubital vein, 90 minutes of dynamic acquisition frames were obtained (4 × 15, 8 × 30, 9 × 60, 2 × 180, 10 × 300, and 2 × 600 s). For each ^11^C-PiB scan, a positron transmission scan or CT scan was obtained for attenuation correction. PET images were reconstructed using an ordered subset expectation maximization algorithm with weighted attenuation. Images were smoothed with a 4 mm Gaussian kernel with scatter correction.

^11^C-PiB data were realigned, and frames from the first 20 minutes of the acquisition were averaged and coregistered to participants’ corresponding structural MRI. Structural MRI was assessed using a 1.5T Siemens Magnetom Avanto scanner at Lawrence Berkeley National Laboratory (T1-weighted MPRAGE images, TR/TE = 2110/3.58 ms, FA = 15°, 1 × 1 × 1 mm resolution). Distribution volume ratios (DVRs) for ^11^C-PiB images were generated with Logan graphical analysis on ^11^C-PiB frames corresponding to 35-90 minutes after injection using a cerebellar gray matter reference region [44, 45]. Global ^11^C-PiB DVR was calculated as a weighted mean across FreeSurfer-derived native space frontal, temporal, parietal, and cingulate cortical regions as in previous studies [29, 30]. Participants were classified as Aβ+ if their global cortical ^11^C-PiB DVR was ≥1.065 in accordance with previous studies [29, 30]. Experimenters were blinded to participants’ Aβ status during data collection.

### Sleep monitoring and EEG analysis

Polysomnography on the nights of the sleep session was recorded using a Grass Technologies Comet XL system (Astro-Med, Inc., West Warwick, RI). This system included a 19-channel EEG placed according to the 10–20 system, electrooculography (EOG) recorded at the left inferior and right superior outer canthi, and electromyography (EMG) on the chin. Reference electrodes were left and right mastoid (A1, A2). Data were digitized at 400 Hz.

Data were scored using a validated automated sleep scoring software [46] and further assured with visual inspection by two trained sleep-scoring professionals (Z.Z. and O.M.) in accordance with standardized criteria [47]. In parallel to sleep scoring, 30-second epochs with major body movements were rejected based on visual inspection. Channels with marked artifact noise were also identified during visual inspection and omitted from subsequent analyses. To reduce artifacts, EEG data were re-referenced from the contralateral mastoids as needed to the average of the mastoids (*N* = 24) or a unilateral mastoid (*N* = 5). Furthermore, for any participant with slow drift artifacts due to sweating (*N* = 27), a semi-automatic, amplitude-based rejection was applied to their EEG data. For each affected participant, a sweat artifact-free, 30-second long epoch from deep sleep (NREM3 stage) was selected based on visual inspection. For each participant and for each EEG channel, the average bandpower between 0.2 and 0.8 Hz in this sweat artifact-free epoch was computed. Further EEG data was rejected on a 5-second basis whenever the bandpower between 0.2 and 0.8 Hz exceeded 1.5 times the individual amplitude of the clean epoch in any of 3 frontal channels (F3, F4, Fz). Frontal channels were specifically selected as the sweat artifact was expressed the strongest in those channels.

Spectral power analysis of artifact-free segments was performed using the validated open-source Python toolbox for sleep analysis (Yet Another Spindle Algorithm, YASA [48]). Data were first downsampled to 100 Hz and filtered between 0.5 and 35 Hz. Next, power spectrum was calculated for NREM and REM sleep stages separately using Welch’s method, median-averaging 4-second intervals with 50% overlap, and employing Hamming windowing. The analyzed frequencies spanned between 0.5 and 35 Hz with a 0.25 Hz bin resolution. Next, bandpower was calculated by integration (area under the curve) for the following frequency ranges: Delta (0.5-4 Hz), Theta (4-8 Hz), Alpha (8-12 Hz), Sigma (12-16 Hz), Beta (16-30 Hz), Gamma (30-35 Hz). Finally, relative power was computed by dividing the bandpowers by the total power observed in the 0.5 - 35 Hz frequency range. Analyses in the current report focused, a priori, on SWA, defined as relative delta spectral power during NREM (NREM2 and NREM3 stages) sleep. Comparing the adaptation night to the experimental night, NREM SWA showed high test-retest reliability (*r* = 0.76, *p* < 0.001).

### Face-name memory task

Next-day memory was assessed using a validated, face-name hippocampal-dependent task sensitive to age and sleep effects [40, 49, 50]. This task has been proven to have good reliability in older adults [51–53]. The task consisted of an encoding phase where 120 face-name pairs were introduced and a recognition test that followed the end of the encoding phase after a 30-minute delay [40].

During the encoding phase, each face-name pair was shown a single time. The task consisted of 4 blocks, each containing 30 trials. Participants were given the opportunity to rest between the blocks. Each block took approximately 5 minutes, with the entire encoding phase taking 20 minutes. For each trial, a fixation was first presented for 0.5 seconds (white background, black Arial font, 24 points, center screen), followed by a face-name pair for 3.5 seconds. To ensure that participants attended to each face-name pair, each pair remained on the screen for an additional 2.75 seconds during which participants had to determine if the name “fit” the face [40]. Participants were instructed to remember each face-name pair for testing. Face stimuli were drawn from a standardized database [54] and were counterbalanced based on gender (male, female) and age group (young: ages 18-49, old ages: 50-90). Names were drawn from the 1990 US census and were counterbalanced for frequency of use in the US population. The presentation order was pseudo-randomized such that no more than four faces of identical gender or age group were presented successively.

The recognition test consisted of 200 trials, with each face/face-name pair tested once. The whole test lasted approximately 45 minutes. Out of the 200 trials, 120 contained faces presented during the encoding, and 80 were novel faces (foils). Foil faces were balanced for age and gender similarly to the encoded material. For each recognition trial, a face was presented on the screen, at the same size and location as presented during encoding. In a self-paced manner, participants first had to answer whether they had seen the face during the prior encoding phase (original) or not (new) by a corresponding keyboard response [40]. Following this, with the face stimulus remaining on the screen, participants were presented with four options to choose from to test associative memory recognition: (1) the original name previously paired with that face (correct ‘hit’ response), (2) a name previously seen before at encoding, but with a different face (incorrect ‘lure’ response), (3) a new name never shown during encoding (incorrect response), or (4) an option “new” rejecting the trial as a foil trial [40]. The four response options appeared horizontally on the screen with the first three always corresponding to names in a randomized order in regards to response type. New (foil) faces were presented with all new names never shown during encoding. As with encoding, faces were presented in a randomized order.

Two distinct d-prime sensitivity memory measures were computed based on the recognition test. Item memory performance was quantified by subtracting the standardized false alarm rate for the faces (the proportion of faces falsely declared to be previously seen out of all the foil trials) from the standardized hit rate for previously encoded faces (the proportion of faces correctly recognized as previously seen out of all the encoded face trials). Associative memory was quantified by subtracting the standardized lure (miss) rate (the proportion of misses, where participants chose the lure names instead of the correct answer out of all previously encoded trials where the participant correctly identified the face as being previously studied) from the standardized hit rate of the face-name pairs (the proportion of faces and names correctly paired out of all previously encoded trials where the participant correctly identified the face as being previously studied).

### Physical activity assessment

Physical activity was quantified using a previously validated questionnaire [41]. A list of 15 leisure-time physical activities (e.g., cycling, dancing, swimming) was provided and participants were asked to report whether they engaged in each of the activities (or any other that was not included in the list). Further, for each activity, information was collected on the frequency (how often during the previous 2 weeks and how many months per year) and duration (time spent per session) of engagement. Frequency and duration information were multiplied using an activity-specific intensity code indicating calorie expenditure [55] and summed to represent the intensity of physical activity (total kilocalories of energy expended) during the past year.

### Memory composite score

Trait-like episodic memory function was measured by a composite score that consisted of short-delay and long-delay (after 20 min) free recall scores of both the California Verbal Learning Test [56] and the Visual Reproduction Test [57]. Individual scores were Z-transformed using mean and SD from the first cognitive session data of a larger sample of BACS participants. The composite score was calculated as the mean of the standardized individual test scores.

### Structural MRI analysis

Selective atrophy within the medial prefrontal cortex (mPFC) has been shown to influence the relationship between sleep and cognitive performance in elderly adults [58, 59]. Therefore, to measure mPFC atrophy, structural MRI scans were obtained from all participants during the sleep session. Structural MRI was assessed using a Siemens Trio 3T scanner. High-resolution T1-weighted MPRAGE images were acquired for every participant (TR/TE = 1900/2.52 ms, FA = 9°, 1 × 1 × 1 mm resolution).

To measure gray matter volume, optimized voxel-based morphometry (VBM) [60] was performed using SPM12 [61] with the Computational Anatomy Toolbox (CAT12) [62] and the Diffeomorphic Anatomical Registration through Exponentiated Lie algebra (DARTEL) [63] toolboxes. Gray matter and white matter segmentations were used to create a study-specific DARTEL template, which was then used to normalize individual brains into MNI space to improve the registration of older brains to the normalized template [64]. Modulated gray matter maps were then smoothed using an 8 mm Gaussian kernel.

To compute gray matter volume in the mPFC, the Anatomical Automatic Labeling repository [65] within the Wake Forest University PickAtlas toolbox [66] was used to generate an anatomically-based ROI. Mean voxelwise gray matter volume within this ROI was extracted using the Marsbar toolbox [67]. Measures of total intracranial volume for each participant were estimated from the sum of gray matter, white matter, and CSF segmentation, and used to adjust mPFC gray matter volumetric measures to account for differences in head size.

### Statistical analysis

Statistical analyses were conducted in R 4.1.0 [68] using the stats, sjPLot, and interaction packages [69, 70]. To compare the global PiB DVR, demographic, sleep, and cognitive measures between the Aβ+ and Aβ- groups, independent, two-sample *t*-tests were used. To test the hypothesis prediction that NREM SWA moderates the effect of Aβ pathology status on memory function, a multiple linear regression was employed with Aβ status, NREM relative delta power, NREM relative delta power by Aβ status interaction, and age, sex, Body Mass Index (BMI), gray matter atrophy, the time difference between the PET and sleep sessions, education, and physical activity as regressors predicting memory performance. Specifically, from this multiple regression, the interaction between NREM relative delta power (SWA) and Aβ status indicated evidence of cognitive reserve. A post-hoc statistical power analysis was performed in GPower 3.1.9.7 to estimate the achieved power of this analysis. To explore regional specificity, multiple linear regressions were computed with relative delta power for each EEG channel separately. To control for multiple testing in the topography analyses, False Discovery Rate (FDR) correction was applied. To assess frequency range and sleep stage specificity, similar multiple linear regressions were employed, by replacing NREM relative delta power with other NREM and REM frequency range spectral power measures. Similar to NREM SWA, the interaction between Aβ status and spectral power measures was evaluated. To control for multiple testing in these non-hypothesis-driven regression results, FDR correction was applied. Further, to differentiate trait and state-like memory benefit effects, a similar multiple regression was applied to evaluate the interaction between Aβ status and NREM SWA predicting the composite memory score that was assessed during a separate session from sleep. For all regressions, cognitive performance and sleep data were excluded on a case-by-case basis, when outside of the 1.5 interquartile range from the first and third quartile across participants in the given Aβ group to ensure normal distribution (see Additional file 1: Table S1 for final sample sizes for each regression by Aβ group).

## Results

Consistent with prior findings [71] and the proposed framework of cognitive reserve under conditions of pathological burden, memory performance was similar in the Aβ+ and Aβ- groups (*t* = -0.17, *p* = 0.86 for item memory and *t* = -0.55, *p* = 0.58 for associative memory, Table 1). These data affirm the relative maintenance of cognitive performance in those with high Aβ burden, indicating the potential presence of cognitive reserve factors explaining a preservation of memory function in the face of such pathology.

We next tested the hypothesis that one novel reserve factor supporting memory preservation across individuals with high Aβ burden was NREM SWA quality. A multiple linear regression model was implemented to assess the interaction between NREM SWA and Aβ status in predicting memory function (Additional file 1: Table S2). Supporting the experimental prediction and a cognitive reserve function, NREM SWA demonstrated a significant interaction with the Aβ status in predicting item memory (*std. β* = 0.64*, p* = 0.042, Fig. 1B). Specifically, the extent of NREM SWA positively predicted superior next-day item memory performance in individuals with high Aβ burden (i.e., those with high cognitive reserve need: *B* = 2.694, 95% CI = [0.472, 4.92], *p* = 0.019), but not in individuals with low Aβ burden (*B* = -0.115, 95% CI = [-1.598, 1.37], *p* = 0.876). Post-hoc power analysis suggested that the study sample size was adequate to detect this interaction effect (achieved power = 0.89, see section “*Achieved power”* in Additional file 1).

To control for possible confounding factors, the multiple regression model also included age, sex, Body Mass Index (BMI), gray matter atrophy (focused on the known NREM SWA-sensitive region of the prefrontal cortex [58]), the time difference between the PET and the sleep session, and known reserve factors of years of education and overall physical activity state marked by total energy expenditure in the past year (in kCal, Additional file 1: Table S2). The interaction between NREM SWA and Aβ status was not only a significant predictor in the model, but notably, it was the strongest predictor of memory performance out of all the factors (*std. β* = 0.64 vs. all other |*std. β|*s *≤ 0.28*, see Additional file 1: Table S2). These findings indicate that the cognitive reserve contribution of NREM SWA is potentially independent of other previously identified factors impacting cognition, including known reserve factors (for further information see section “*Interaction of NREM SWA with previously identified cognitive reserve factors, education and physical activity”* in Additional file 1).

To determine EEG frequency specificity, associations between memory performance and NREM EEG spectral power in other frequency ranges were examined by employing multiple regression models similar to the NREM SWA model but replacing the delta frequency range spectral power with other frequencies. The interaction between Aβ status and spectral power was not significant in any other frequency range, indicating the selectivity of the NREM SWA frequency range (Additional file 1: Table S3). In addition, and indicating sleep-stage selectivity, no significant interactions between Aβ status and EEG spectral power in REM sleep were evident after multiple comparison correction (Additional file 1: Table S3). These findings demonstrate both oscillation frequency and sleep stage specificity regarding the association of sleep with cognitive reserve.

The above analyses established an association between cognitive function and NREM SWA averaged across the entire scalp. However, studies in older adults have demonstrated regionally specific EEG channel associations of sleep with memory function, dominant over prefrontal cortex regions [27, 59, 72]. Motivated by these local cortical relationships, we next sought to explore whether the associations between NREM SWA and memory preservation in Aβ+ individuals were common in strength across all EEG derivations, or demonstrated regional specificity. Examining the topography in the Aβ+ group, NREM SWA showed largely similar strength distribution over the entire scalp (Fig. 1C).

Finally, to test whether sleep’s support of memory function was a trait-like or a state-like effect, (i.e., general or specific to performance the next day), a multiple regression analysis was performed, identical to that above predicting next-day memory function, but with the composite memory score that was measured in a separate session from NREM SWA. Consistent with a state-like specificity of cognitive reserve function, this multiple regression, where sleep and memory function were assessed in separate sessions, did not reveal a significant interaction between NREM SWA and Aβ status in predicting memory (*std. β* = 0.11*, p* = 0.473).

## Discussion

Together, these findings are consistent with the proposal that NREM SWA represents a novel cognitive reserve factor supporting next-day memory function under conditions of high resilience need, here, the circumstance of high Aβ burden. Adding specificity, no other significant positive predictive memory associations emerged with other spectral power characteristics of NREM or REM sleep in the Aβ+ group.

Furthermore, the association between NREM SWA and next-day memory function was significant when accounting for covariates, including age, sex, BMI, prefrontal gray matter atrophy, and those previously linked to resilience, i.e., education and physical activity. The latter finding might be reflective of sleep as an independent factor supporting cognitive reserve. This would suggest that combining the appliance of multiple resilience factors, including sleep, would be additive in their cognitive reserve benefit, rather than redundant.

Mechanistically, our findings may be accounted for by one or more of three non-mutually exclusive frameworks. The first is the synaptic homeostasis hypothesis [73–75], which suggests that learning during wakefulness increases synaptic strength in learning-dependent networks, while NREM SWA downscales and thus re-normalizes synaptic strength, thereby restoring optimal learning. This becomes relevant for the current findings considering the aberrant conditions of synaptic potentiation and hippocampal hyperactivity that have been linked to high Aβ burden detectable already in early disease stages [76–79]. Therefore, under conditions of high Aβ burden (Aβ+ participants), NREM SWA may play an especially critical role in the management of Aβ-related synaptic over-potentiation (e.g., within the hippocampus), with the extent of NREM SWA therefore predicting the restored efficiency of next-day learning.

A second explanatory mechanism concerns the hippocampal-neocortical transformation of memory, within which there is a recognized role for NREM SWA in promoting superior memory consolidation and the restoration of hippocampal learning the next day [80–83]. Specifically, newly acquired memory traces are further processed during SWS and through a hippocampal-neocortical dialogue become increasingly hippocampal-independent [84, 85]. Indeed, preventing sleep and specifically, SWS, causally negates this transaction, and results in decreased next-day hippocampal encoding ability [26, 86–88]. Since high Aβ burden is associated with hippocampal hyperactivity [76, 78, 89], NREM SWA may be especially necessary for hippocampal-neocortical transformation in those with greater AD pathology burden to restore the next-day functional activity state of the hippocampus and thus associated learning.

A third, novel mechanism concerns glymphatic clearance of metabolic solutes from the brain during NREM SWS [90, 91], the glymphatic influx potency of which is predicted by NREM SWA [92]. Superior glymphatic clearance and higher cognitive functioning have been reported in older human adults [93] and rodents [94–96]. Within this proposed framework, those with a high Aβ burden (Aβ+) would benefit more from the mediating glymphatic-enhancing benefit of NREM SWA, expressed in superior cognitive reserve, relative to those with lower Aβ burden, as observed in the current study.

Beyond mechanism, the current findings have therapeutic implications. Unlike many of the previously identified cognitive reserve factors (such as years of education, prior occupation(s), or size of social network [14–18, 97]), sleep is a modifiable target. As a result, NREM SWA may represent a novel therapeutic possibility aiding cognitive reserve. Such a potential is present-day viable, considering that sensory (auditory tones, odors), transcranial electrical, and magnetic stimulation methods have all been demonstrated to increase NREM SWA [24, 25, 98–101]. This is similarly true for less technologically challenging methods, such as at-home possible body temperature manipulation [102, 103]. Harnessing these existing tools for the augmentation of NREM SWA could potentially aid in the preservation of cognitive function in the face of AD pathology, both in the present moment and, with repeated application, longitudinally.

Several testable hypotheses now emerge as next steps following the first establishment of a cognitive reserve association between NREM SWA and memory under high Aβ burden. First, does the enhancement of NREM SWA (described earlier) lead to causal improvement in cognition expressly under conditions of high Aβ burden, and less so under non-pathological demanding conditions of low Aβ load? Second, do those who maintain higher NREM SWA quality in the long-term (years) demonstrate preservation of cognitive function longitudinally, *even with* the continued escalation of Aβ burden? Third, does sleep confer a similar cognitive reserve function in the face of the other common pathology components of the Alzheimer’s disease cascade, such as tau burden (that was not testable in the current study)? Notably, tau burden has recently been shown to correlate not with NREM SWA, but instead, to be linked with the precise coupling of slow waves with sleep spindle activity [31]. Therefore, it is possible that other sleep oscillations and/or sleep stages or even macrostructure features (e.g., sleep efficiency [28]) offer cognitive reserve in response to different AD pathological features, or at different stages during the AD pathological cascade.

Finally, our study must be appreciated within the context of important limitations. First, our findings describe an association between sleep and next-day memory performance but do not establish directional causality. Second, it is possible that other, unmeasured factors, such as tau pathology or structural brain changes (e.g., white matter atrophy), may explain additional variance in sleep-dependent cognitive reserve expressed. Third, and relatedly, participants in the study consisted of a relatively healthy cohort that limits generalizability to the entire elderly population or those with AD.

## Conclusions

In summary, we offer evidence that one novel and previously unexplored cognitive reserve factor in the face of Aβ burden is sleep, and specifically the quality of NREM SWS. Of therapeutic importance, and unlike many other cognitive reserve factors identified to date, sleep may represent a novel modifiable reserve factor and thus a promising treatment target.

## Supplementary Information

**Additional file 1: **Results section “*Association between Aβ burden and spectral power in the delta frequency range”*. **Figure S1.** Association of Aβ burden with relative slow and fast delta spectral power in the Aβ+ and Aβ- groups. Results sections *“Achieved power”* and *“Interaction of NREM SWA with previously identified cognitive reserve factors, education and physical activity”.* **Table S1.** Final sample sizes and number of outliers per Aβ group in each regression. **Table S2.** Regression analysis results for predicting item memory. **Table S3.** Estimates and significance levels of the interaction terms of Aβ status and NREM and REM spectral power in multiple regression models predicting memory.

## Data Availability

The data that support the findings of this study are available from the corresponding author upon reasonable request and approval following university review.

## Abbreviations

AD: Alzheimer’s disease
CI: Confidence interval
Aβ: β-amyloid
SWS: Slow wave sleep
SWA: Slow wave activity
BMI: Body Mass Index
DVR: Distribution volume ratio
PiB: Pittsburgh compound B
EEG: Electroencephalography
EOG: Electrooculography
EMG: Electromyography
FDR: False Discovery Rate
FFT: Fast Fourier Transform
MRI: Magnetic resonance imaging
NREM: Non-Rapid eye movement
REM: Rapid eye movement
PET: Positron Emission Tomography
PSG: Polysomnography
BACS: Berkeley Aging Cohort Study
WASO: Wake after sleep onset
mPFC: Medial prefrontal cortex
MMSE: Mini-Mental State Examination
