# Supplementary material for: NREM sleep as a novel protective cognitive reserve factor in the face of Alzheimer's disease pathology
Source: BMC Med. 2023 May 3;21:156. doi: 10.1186/s12916-023-02811-z (PMC10155344; doi:10.1186/s12916-023-02811-z)
Supplement: Supplementary file 1 — Additional file 1: Results section “Association between Aβ burden and spectral power in the delta frequency range”. Figure S1. Association of Aβ burden with relative slow and fast delta spectral power in the Aβ+ and Aβ- groups. Results sections “Achieved power” and “Interaction of NREM SWA with previously identified cognitive reserve factors, education and physical activity”. Table S1. Final sample sizes and number of outliers per Aβ group in each regression. Table S2. Regression analysis results for predicting item memory. Table S3. Estimates and significance levels of the interaction terms of Aβ status and NREM and REM spectral power in multiple regression models predicting memory. [file 12916_2023_2811_MOESM1_ESM.docx]

**Additional file for the manuscript titled**

NREM sleep as a novel protective cognitive reserve factor in the face of Alzheimer's disease pathology

Zsófia Zavecz, Vyoma D. Shah, Olivia G. Murillo, Raphael Vallat, Bryce A. Mander, Joseph R. Winer, William J. Jagust, Matthew P. Walker

**Results**

*Association between Aβ burden and spectral power in the delta frequency range*

Relative delta spectral power was higher in the Aβ+ group compared to the Aβ- group in the entire delta frequency range (0.5-4 Hz, see Table 1 in the main text). This result may seem counterintuitive at first, considering reports of Aβ being associated with impaired NREM slow wave activity (SWA) [27,31]. However, this result of higher relative spectral power in the entire delta range in the Aβ+ group is in line with previous research and can be understood when considering the nuanced difference on the basis of the frequency range of delta spectral power (0.5-1 Hz slow delta vs. 1-4 Hz fast delta), as well as Aβ status (Aβ- vs Aβ+).

Regarding the frequency range, spectral power in the fast delta frequency range (1-4 Hz)—which comprises the large majority of the calculated full delta spectral range (0.5-4 Hz)—has previously been shown to scale progressively higher, the greater the degree of the Aβ burden is [27]. Consistent with this past finding, relative spectral power in the fast delta frequency range was significantly higher in the Aβ+ group than in the Aβ- group in our sample (*t* = -3.80, *p* = 0.0003). However, there was no significant difference between the groups in relative power in the slow delta frequency range (0.5-1 Hz, *t* = -0.82, *p* = 0.41).

The association between Aβ burden and delta power was further altered by Aβ status (**Figure S1**). In the Aβ- group there was no association between relative fast or slow delta power and Aβ burden (all *p*s > 0.94). However, in the Aβ+ group, Aβ burden exhibited a significant negative association with slow delta power (*B* = -1.37, *p* = 0.03) and a positive association with fast delta power (*B* = 1.82, *p* = 0.11).

Together, these results show that fast delta power—which comprises most of the full-range delta power band—increases with greater Aβ burden in Aβ+ individuals (while slow delta does the opposite). This result helps explain the counterinitiative finding of greater overall broad-spectrum delta power we report in the Aβ+ group, relative to Aβ- group. While speculative, such findings may indicate that the decreased slow delta activity (the neural mechanism of which is different from that of fast delta [32,33]) caused by Aβ may result in a homeostatic compensation mechanism that results in the brain offsetting such decreased quality by increasing the amount of overall delta activity, as was present in the Aβ+ group.


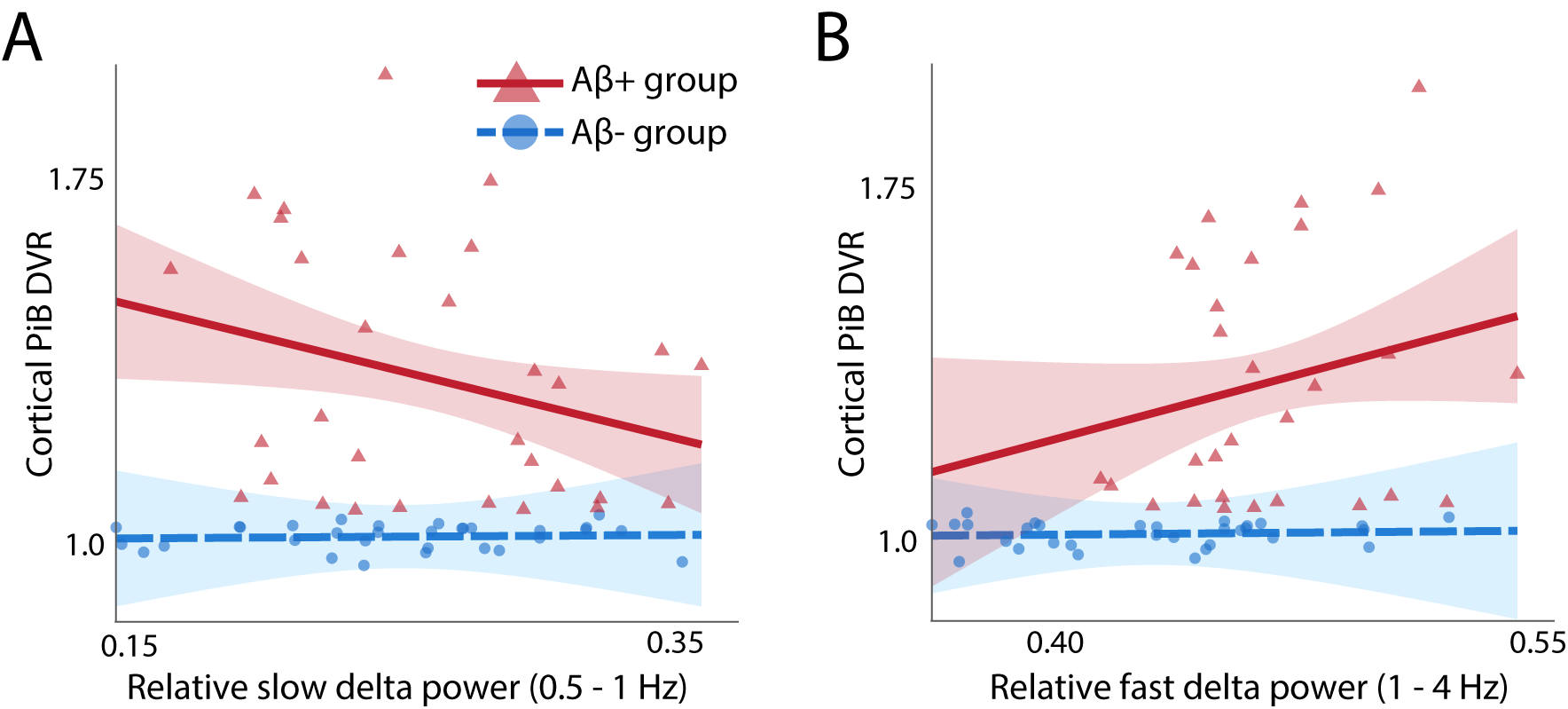


**Figure S1. Association of Aβ burden with relative slow (A) and fast (B) delta spectral power in the Aβ+ (red) and Aβ- (blue) groups.** Selectively in the Aβ+ group, relative slow delta power (0.5-1 Hz) decreased with Aβ burden, whereas relative fast delta power (1-4 Hz) increased (regression line, 95% CI interval, and individual subject data points illustrated in red and blue for each respective group).

*Achieved power*

Achieved power was calculated using the Linear bivariate regression: Two groups, difference between slopes option with the sample size of 54 (30 vs. 24), slope difference of 2.81 (as observed between the Aβ+ and Aβ- group), and an alpha level of 0.05. This analysis indicated an achieved power of 0.89, thus, the sample size available in the study was adequate for the main objective of the study.

*Interaction of NREM SWA with previously identified cognitive reserve factors, education and physical activity*

To assess the nature of the relationship between cognitive reserve factors, we conducted further linear regression models in the Aβ+ group investigating whether education or physical activity would significantly interact with NREM SWA in predicting item memory. The same covariates were controlled for as in all regression models (age, sex, BMI, gray matter atrophy, the time difference between the PET and sleep sessions, education, and physical activity). There was no significant interaction between NREM SWA and education (*p* = 0.76) or physical activity (*p* = 0.49) in predicting memory function. This result further reinforces the independence of these cognitive reserve factors in this relatively healthy older adult population.

**Table S1.** Final sample sizes and number of outliers per Aβ group in each regression.

| Regression | Aβ- group | | Aβ+ group | |
| --- | --- | --- | --- | --- |
|  | *N* | *N*_outliers_ | *N* | *N*_outliers_ |
| Hypothesis-driven |  |  |  |  |
| Item memory NREM relative delta power | 30 | 1 | 24 | 2 |
| Associative memory NREM relative delta power | 31 | 0 | 26 | 1 |
|  |  |  |  |  |
| Exploratory |  |  |  |  |
| Item memory theta NREM relative theta power | 28 | 3 | 25 | 1 |
| Associative memory NREM relative theta power | 29 | 2 | 27 | 0 |
| Item memory NREM relative alpha power | 30 | 1 | 24 | 2 |
| Associative memory NREM relative alpha power | 31 | 0 | 26 | 1 |
| Item memory NREM relative sigma power | 27 | 4 | 24 | 2 |
| Associative memory NREM relative sigma power | 28 | 3 | 26 | 1 |
| Item memory NREM relative beta power | 29 | 2 | 24 | 2 |
| Associative memory NREM relative beta power | 30 | 1 | 26 | 1 |
| Item memory NREM relative gamma power | 30 | 1 | 21 | 5 |
| Associative memory NREM relative gamma power | 31 | 0 | 23 | 4 |
| Memory composite score NREM relative delta power | 31 | 0 | 28 | 1 |
| Item memory REM relative delta power | 30 | 1 | 23 | 2 |
| Associative memory REM relative delta power | 31 | 0 | 25 | 1 |
| Item memory REM relative theta power | 29 | 2 | 23 | 2 |
| Associative memory REM relative theta power | 30 | 1 | 25 | 1 |
| Item memory REM relative alpha power | 29 | 1 | 24 | 2 |
| Associative memory REM relative alpha power | 30 | 0 | 26 | 1 |
| Item memory REM relative sigma power | 30 | 1 | 23 | 2 |
| Associative memory REM relative sigma power | 31 | 0 | 25 | 1 |
| Item memory REM relative beta power | 29 | 2 | 22 | 3 |
| Associative memory REM relative beta power | 30 | 1 | 24 | 2 |
| Item memory REM relative gamma power | 29 | 2 | 22 | 3 |
| Associative memory REM relative gamma power | 30 | 1 | 24 | 2 |
| Memory composite score REM relative delta power | 31 | 0 | 28 | 1 |

**Table S2.** Regression analysis results for predicting item memory

|  | *B* | 95% CI | *p* | *Std. β* | *F* | *df* | *p* | adj. *R*^2^ |
| --- | --- | --- | --- | --- | --- | --- | --- | --- |
| Overall model |  |  |  |  | 3.11 | 10, 43 | 0.03 | 0.20 |
| NREM SWA | -0.12 | [-1.60 – 1.37] | 0.876 | -0.03 |  |  |  |  |
| Aβ status | -1.96 | [-3.91 – 0.00] | 0.050 | 0.05 |  |  |  |  |
| Age (years) | -0.02 | [-0.04 – 0.00] | 0.084 | -0.26 |  |  |  |  |
| Sex | -0.08 | [-0.26 – 0.10] | 0.380 | -0.12 |  |  |  |  |
| BMI | -0.02 | [-0.04 – 0.01] | 0.134 | -0.20 |  |  |  |  |
| Prefrontal gray matter atrophy | -0.02 | [-0.10 – 0.05] | 0.537 | -0.09 |  |  |  |  |
| Time between PET and PSG sessions | -0.00 | [-0.00 – 0.00] | 0.569 | -0.08 |  |  |  |  |
| Education (years) | 0.05 | [-0.00 – 0.10] | 0.051 | 0.28 |  |  |  |  |
| Physical activity (kCal expenditure past year) | 0.00 | [-0.00 – 0.00] | 0.514 | 0.09 |  |  |  |  |
| NREM SWA * Aβ status | 2.81 | [0.10 – 5.52] | 0.042 | 0.64 |  |  |  |  |

*Note* The dependent variable was item memory performance.

BMI = Body Mass Index, SWA = slow wave activity

**Table S3.** Estimates and significance levels of the interaction terms of Aβ status and NREM and REM spectral power in multiple regression models predicting memory

|  | Item memory | |  | | Associative memory | | | |
| --- | --- | --- | --- | --- | --- | --- | --- | --- |
|  | *Std. β* | *p* | | FDR *p* | | *Std. β* | *p* | FDR *p* |
| Hypothesis-driven |  |  | |  | |  |  |  |
| NREM relative delta power | 0.64 | 0.042 | | N/A | | -0.03 | 0.983 | N/A |
|  |  |  | |  | |  |  |  |
| Exploratory |  |  | |  | |  |  |  |
| NREM relative theta power | -0.00 | 0.990 | | 0.990 | | -0.01 | 0.942 | 0.963 |
| NREM relative alpha power | -0.36 | 0.058 | | 0.213 | | -0.01 | 0.963 | 0.963 |
| NREM relative sigma power | -0.17 | 0.281 | | 0.490 | | -0.11 | 0.448 | 0.700 |
| NREM relative beta power | -0.12 | 0.498 | | 0.684 | | -0.14 | 0.399 | 0.700 |
| NREM relative gamma power | -0.04 | 0.834 | | 0.917 | | -0.20 | 0.301 | 0.700 |
| REM relative delta power | 0.16 | 0.312 | | 0.490 | | -0.14 | 0.367 | 0.700 |
| REM relative theta power | -0.05 | 0.741 | | 0.906 | | 0.09 | 0.526 | 0.700 |
| REM relative alpha power | -0.16 | 0.272 | | 0.490 | | 0.08 | 0.573 | 0.700 |
| REM relative sigma power | -0.30 | 0.058 | | 0.213 | | -0.25 | 0.117 | 0.700 |
| REM relative beta power | -0.40 | 0.039 | | 0.213 | | -0.19 | 0.338 | 0.700 |
| REM relative gamma power | -0.22 | 0.257 | | 0.490 | | -0.13 | 0.518 | 0.700 |

*Note* Results of regressions predicting memory, with age, sex, BMI, gray matter atrophy, education, physical activity, the time difference between the PET and sleep sessions, Aβ status, spectral power, and the interaction between Aβ status and spectral power as regressors.
